# Second Annual Meeting of the North Dakota Medical Association

**Published:** 1894-02

**Authors:** T. D. Hinebauch

**Affiliations:** Secretary, Fargo, N. D.


### SECOND ANNUAL MEETING OF THE NORTH DAKOTA MEDICAL ASSOCIATION.

The North Dakota Veterinary Medical Association met at Hillsboro, December 30th, 1893, Doctor Crewe, President, in the chair.

After reading of the minutes by the Secretary, Dr. Hinebauch moved that Article III of the Constitution, which reads as follows, "Members of this organization shall consist of graduates and non-graduates as recommended by the Board of Censors. Such applicants as are non-graduates shall pass an examination which shall be deemed sufficient by the Board of Examiners appointed for the purpose; examination to take place at the first annual meeting after application has been made," be amended as follows: "Members of this organization shall consist of graduates of recognized veterinary colleges."

Under the Constitution and By-Laws the amendment cannot come up for final vote until the next annual meeting. It was the sense of the Association, however, that the change was a desirable one.

Dr. Taylor then presented a paper on glandered horses, of which the following is a synopsis:

"Every intelligent owner of a horse is more or less acquainted with that loathsome disease known as glanders, either by tradition or experience. He is aware that it is both contagious and fatal not only to horses, but also to mankind. The fact that the disease has to this day baffled all treatment is sufficient evidence why its detection in a stable is always looked upon as serious. If the disease always assumed the same character, if every glandered horse presented the three special symptoms essentially belonging to it, viz.: sticky and bloody discharge from the nose, hard, painless, inflamed and swollen glands of the jaws, and above all the peculiar and characteristic ulcerations of the mucous membranes of the nasal cavities and chambers, there would be no difficulty in recognizing it, and condemning the animal affected as most dangerous. It is found, however, that horses which suffer from glanders in this country, where the climate is especially dry, that cases are very rare which exhibit any two of these symptoms. At the same time cases do exist which do not exhibit even one of the symptoms, thus making it very difficult to diagnose.

"Animals affected in the latter way are fully as dangerous in transmitting the disease as those which show the symptoms peculiar to the disease; they live longer, and are more apt to pass from one farm to another, thus coming in contact with more horses, and eventually producing a wider spread of the disease than where the typical acute form exists.

"When the disease is once detected there should be no hesitation in destroying the animal. even if doubt exists in the owner's mind regarding the disease; he should not hesitate to call in a properly qualified veterinarian, in order that he may be advised with regard to the true condition which exists."

The discussion which followed elicited the fact that a number of appointments had been made in the different veterinary districts of the State of men who are neither qualified by experience or education to fill the position of District Veterinarian. Many horses undoubtedly have been destroyed for glanders which did not have the disease at all; while on the other hand many which did show unmistakable symptoms of the disease have been allowed to go at large. scattering the disease over wide areas.

Dr. Farmer presented the subject, "Pleuro-pneumonia of a contagious form among horses," but as he had no regularly-prepared paper, his subject was not discussed as thoroughly as it would have been had he placed his observations in writing. Right here I would again like to call the attention of members of this Association, as well as of other associations, to the fact that all papers and communications should invariably be presented in writing, so that the Secretary can report a proper synopsis of them to the veterinary journals.

After the reading of the papers, Dr. D. Fisher, of Grandin, made application, and on vote was admitted to membership in the Association. It was also moved that the Secretary admit all applicants for membership in the Association who can present credentials that are satisfactory. The Association, although but in its second year, is upon good footing, increasing in numbers, and at the present time but three graduates live in the State who do not belong.

T. D. HINEBAUCH, *Secretary*, Fargo, N. D.

